# The Release of Organic Acids and Low Molecular Weight Carbohydrates from Matcha Tea After In Vitro Digestion

**DOI:** 10.3390/nu16234058

**Published:** 2024-11-26

**Authors:** Jiří Nekvapil, Daniela Sumczynski, Richardos Nikolaos Salek, Martina Bučková

**Affiliations:** 1 Department of Food Analysis and Chemistry, Faculty of Technology, Tomas Bata University in Zlín, Vavrečkova 5669, 760 01 Zlín, Czech Republic; 2 Department of Food Technology, Faculty of Technology, Tomas Bata University in Zlín, Vavrečkova 5669, 760 01 Zlín, Czech Republic

**Keywords:** bioactive compound, foods, matcha, *Camellia sinensis*, green tea, low molecular weight carbohydrate, organic acid, in vitro digestion

## Abstract

Background/Objectives: This study tested the influence of in vitro digestion on the release of organic acids and low molecular weight saccharides of matcha. Methods: The concentrations of analytes in the raw and undigested portion of matcha were measured using HPLC with spectrometric and refractometric detection to establish their residual values after a two-step enzymatic digestion that was finally presented as a retention factor. Results: It was established that dry matter digestibility values after simulated gastric and both gastric and intestinal phases were 67.3 and 85.9%, respectively. Native matcha, citric acid (44.8 mg/g), malic acid (32.2 mg/g), trehalose (36.1 mg/g), and L-arabinose (8.20 mg/g) reached the highest values and were predominant, whereas D-fructose, xylose, maltose, and saccharose were not detected. Regarding gastric phase digestion, succinic and malic acids, trehalose and D-glucose were the worst-releasing compounds and their remaining factors reached 34, 19, 18, and 50%, respectively, whereas L-arabinose was completely released. Focusing on gastric and small intestinal digestion, the least-releasing compounds of matcha tea leaves were succinic acid and trehalose, with their retention factors at 7 and 13%, which can proceed with the leaf matrix to the large intestine. Conclusions: Malic, oxalic, and citric acids, the carbohydrates D-glucose, L-arabinose, and L-rhamnose, are almost entirely released from matcha tea during digestion in the stomach and small intestine and can be available for absorption in the small intestine. In the measurement of oxalic acid, considering that the process of shading tea leaves increases the concentration of this acid and its retention factor value is too small, it would be appropriate in the future to evaluate the recommended maximum daily intake of matcha tea for people sensitive to the formation of urinal stones.

## 1. Introduction

Depending on the degree of leaf oxidation, the most common types of teas are green, oolong, and black tea [1,2]. Matcha tea is a variety of powdered Tencha green tea (*Camellia sinensis*) that differs from traditional green tea in conditions during cultivation, when it is characteristically prepared from green tea leaves grown under the shade technique that are steamed, dried, and finally ground on several millstones [3,4,5,6,7,8]. The processes of preparing and consuming matcha tea also differ from other teas. Generally, green tea is consumed as a water extract of the leaves, whereas in matcha tea, the ground leaves are covered with water, and the whole leaves are consumed [9].

The effect of the different processing and consumption methods leads to the ingestion of more significant amounts of bioactive substances occurring in tea, e.g., polyphenols, amino acids, saponins, chlorophyll, L-theanine, and caffeine. Most of these compounds have been declared to positively affect human health [5,10,11]. The health effects associated with drinking green tea have been reported to comprise protection against cancer, lowering the risk of cardiovascular disease and stroke, cognitive dysfunction, allergy relief effects, and preventive effects on metabolic syndrome, reducing the development of mortality, and three important aspects of death, such as heart disease, respiratory diseases, and cerebrovascular disease [5,9,11,12,13].

Generally, carbohydrates are produced in plant cells through the process of photosynthesis. In particular, carbohydrates serve plants for the growth and development of cellular structures; they are a source of energy, and many of them are used by cells for the biosynthesis of lipids, proteins, and polysaccharides [14]. *Camellia sinensis* raw leaves are composed of carbohydrates in the range of 4–7% of dry weight (DW) [15]. It is a generally known fact that green tea shoots constitute 4–10% of polysaccharides, 6–8% of cellulose, and 3–5% of low molecular weight carbohydrates [15]. Monosaccharides such as D-arabinose, D-galactose, D-glucose, L-rhamnose, D-fructose, and oligosaccharides such as maltose, sucrose, raffinose, lactose, and stachyose were noticeably determined in green tea [12,16]. Moreover, the α-galactooligosaccharides such as raffinose and stachyose are well known to serve as prebiotics [12], and myo-inositol contributes to female reproductive health [15].

Organic acids are intermediate products of carbohydrates in metabolic pathways, such as the Krebs cycle and the shikimic acid pathway, and they play a role in maintaining cell osmotic pressure [17]. Tea leaves are a significant source of malic and oxalic acids along with citric, isocitric, and succinic acids. For instance, oxalic and citric acids are co-responsible for the quality of green tea leaves, and succinic and citric acids enhance the umami taste of glutamic acid in matcha [8,17,18]. More profoundly, shade treatment decreased the content of quinic and succinic acid but increased the content of malic and citric acids in matcha tea [11]. Both shikimic and quinic acids enter the biosynthesis of the polyphenols. The content of quinic acid present in the stems has also been reported to be one of the predictor factors prolonging tea quality and is recognized to be used as an anti-depressive and cognition-improving substance [11]. On the contrary, the total amount of organic acids such as succinic, oxalic, malic, lactic, and citric acids, etc., increases dominantly after withering, rolling, and fermentation, when black tea is produced [17].

Metabolic processes in the intestinal tract affect the release and absorption of individual nutrients of the food matrix. Therefore, consuming food rich in individual nutrients does not necessarily mean that more of these compounds will be absorbed into the digestive tract. The effect of matcha tea polyphenols on human organism has been addressed, including the digestibility of phenolics [19]. Both bioavailability and bioaccessibility are important factors for determining the efficiency of the nutrient intake from meals and its further total nutritional value [20,21]. In addition, innovative research has been conducted to analyze the percentage of solid undigested proportions of plant food to evaluate the rest of the bioactive compounds after the in vitro digestion process. This factor represents the amount of analyte retained in the undigested matrix expressed as percentage [19,22].

To the best of our knowledge, we are unaware of any studies that have addressed the influence of digestibility under in vitro conditions on low molecular weight carbohydrates and organic acids after digestion in the stomach, and subsequently in the small intestine simulated under in vitro conditions. Therefore, a simulated in vitro digestion process provided by pepsin and pancreatin enzymes under human body temperature was applied. The aim of this research was to assess the contents of individual low molecular weight carbohydrates and organic acids in the native and undigested portions of matcha powders using a high-performance liquid chromatography method with the spectrophotometric and refractometric detection. Moreover, the appropriate retention factors (RF) of the analyzed compounds in the undigested portions were expressed as well.

## 2. Materials and Methods

### 2.1. Chemicals

Pepsin with an enzymatic activity of 2000 FIP-U/g, and a mixture of pancreatic enzymes such as protease, amylase, and lipase with activities of 350, 7500, and 6000 FIG-U/g, respectively, were purchased from Carl Roth and Merck (Karlsruhe and Darmstadt, Germany). HPLC standards in purity of ≥99.5% (L-arabinose, L-rhamnose, D-glucose, D-fructose, xylose, maltose, saccharose, trehalose, oxalic, succinic, malic and citric acids) were acquired from Sigma-Aldrich (St. Louis, MI, USA). Acetone, HCl, KH_2_PO_4_, and Na_2_HPO_4_ 12 H_2_O were provided from Sigma-Aldrich (St. Louis, MI, USA). In addition, KH_2_PO_4_, methanol, acetone, HCl, and Na_2_HPO_4_×12 H_2_O were supported by Penta (Prague, Czech Republic). Redistilled water was supplied by a Purelab Classic Elga water system (Labwater, London, UK).

### 2.2. Matcha Tea Samples

The matcha samples tested consisted of five high-quality organic Japanese and Chinese powders, and a matcha tea (*Camellia sinensis*), produced from Tencha leaves. Five matcha tea samples were selected for analysis as follows: premium Organis matcha tea, premium Allnature organic matcha tea, Imbio matcha tea (all originating in China), Iswari organic matcha powder, and Natu matcha tea (both originating in Japan). All were sold with expiration dates in 2024. They were kept in the original sacks (0.070 to 0.25 kg) without access to sunlight under the control temperature of about 23 ± 2 °C for a maximum of two months.

### 2.3. Digestion Process Simulated In Vitro

**Figure 1:**
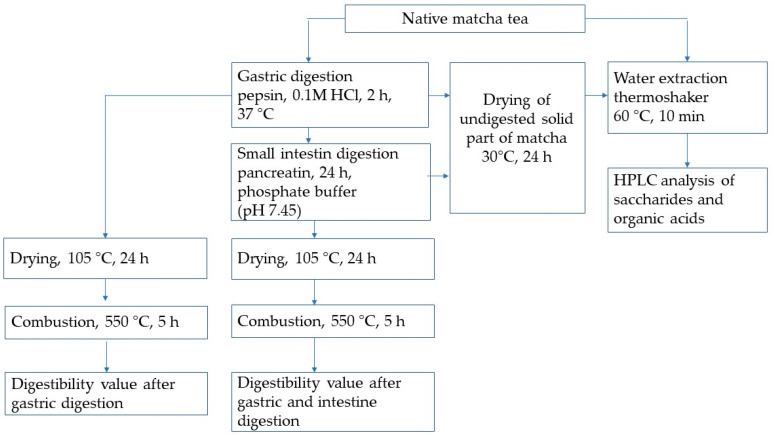
Scheme of the experiment.

The gastrointestinal process, simulated under in vitro conditions, including gastric and intestinal phases and determinations of ash and dry matter content, were determined according to Koláčková [19] with a slight modification (Figure 1).

The in vitro digestibility value of the powder form of matcha tea was firstly determined using pepsin and pancreatin enzymes in an incubator (Daisy^II^, Ankom Technology, Macedon, NY, USA). The matcha tea sample (0.25 g) was weighed and sealed by impulse sealer (KF-200H, Penta Servis, Holice, Czech Republic) in digestion sacks (F57 type, Ankom Technology, Macedon, NY, USA).

To model the gastric condition, the experimental flask was infused by 1.7 L of 0.1 M HCl containing pepsin (0.63 g). Consequently, the samples were exposed for 2 h at 37 °C and then washed by redistilled water. To simulate small intestine conditions, phosphate buffer (consisting of Na_2_HPO_4_×12 H_2_O and KH_2_PO_4_, pH 7.45) and a mixture of pancreatin enzymes (3.0 g) were dissolved in 1.7 L of redistilled water and added to the incubation flask. After a twenty-four hours incubation at 37 °C, the samples were treated with redistilled water followed by drying at 105 °C for 24 h, and weighting.

In the final step of the analysis, the sacks containing the rests of the matcha tea were burned in a muffle oven (LM112.10, Veb Elektro, Berlin, Germany) at 550 °C for 5 h, so they could be cooled in an exicator and weighed. All assessments were repeated as three independent experiments. In vitro dry matter digestibility value (DMD) was calculated using Formulas (1)–(5):

DMD = 100 − [(100 × DMR)/(m_2_ × DM)] (1)

DMR = m_3_ − m_1_ × c_1_ (2)

DM = (DW × m_s_)/100 (3)

c_1_ = m_s_/m_1_ (4)

c_2_ = m_p_/m_1_ (5)

where the appropriate values indicate the following: DMD (dry matter digestibility, %), DMR (amount of the sample without the sack after the digestion, g), DM (dry weight of the sample, g), DW (dry weight of the sample presented in %), m_s_ (the sample amount, g), c_1_ (the correction of the weight of the sack after the incubation, g), c_2_ (the correction of the weight of the sack after the burning, g), m_p_ (the amount of ash from the empty correction sack, g), m_1_ (the weight of the empty bag, g), m_2_ (the sample amount, g), m_3_ (the weight of the dried bag with the sample after the incubation, g).

#### Preparation of Undigested Portions of Matcha Leaves

To obtain both forms of undigested portions of matcha, the digestibility assessment was determined as follows: (i) after stomach incubation, when the undigested part of the matcha sample was dried at 30 °C for 24 h, (ii) after stomach and small intestine digestion, when the undigested part of the matcha sample was dried at 30 °C for 24 h (Figure 1). These experiments were repeated three times. The undigested residue of matcha obtained in solid form was extracted following the methodology described in Section 2.4.

### 2.4. Extraction of Low Molecular Weight Saccharides and Organic Acids

Extracts from matcha tea samples were prepared in the same way to determine free carbohydrates and organic acids. The sample extracts were divided into three groups as follows: (i) native matcha sample, in which the digestion process was not carried out; (ii) undigested part of the matcha sample, in which in vitro digestion was terminated in the stomach; (iii) undigested part of the matcha sample, in which in vitro digestion was performed in both the stomach and the small intestine. A sample of 0.2 g of powder matcha from each treatment group was weighed in 2 mL plastic Eppendorf tubes with 2 mL of redistilled water, and the microtube content was mixed. Subsequently, the tube was placed in a TS-100 thermoshaker (Biosan, Riga, Latvia) for 10 min at 60 °C to extract carbohydrates and organic acids. Consequently, the sample was centrifuged for 10 min at 23,000× *g* (Velocity 13μ, Dynamica Scientific Ltd., London, UK) and filtered through a nylon syringe filter (13 mm × 0.45 μm). The filtrate thus prepared was processed for HPLC analysis [11,23].

### 2.5. Determination of Individual Organic Acids Using High-Performance Liquid Chromatography

The organic acid profile (oxalic, succinic, and malic and citric acids) was tested using an HPLC Dionex Ultimate 3000 liquid chromatogram system equipped by DAD detector (DAD-3000 RS, Thermo Scientific Waltham, MA, USA) with minor modifications [24]. Organic acids were separated on a Phenomenex Synergi Hydro-RP C18 column (250 × 4.6 mm; 4 μm, Phenomenex; Torrance, CA, USA). Twenty µL of sample extract was applied into the column. Regarding the gradient mode, two mobile phases were used: (A) 20 mM of potassium dihydrogen phosphate and (B) methanol. The gradient elution had the following settings: 0% of B at 0–2.5 min; 0–30% of B between 2.5 and 2.6 min; 30% of B between 2.6 and 2.9 min; 30–0% of B between 2.9 and 3.0 min; 0% of B between 3.0 and 10 min. The mobile phase flow rate was set at 1 mL/min, the column temperature was adjusted at 60 °C, and the wavelength of 210 nm for recording the chromatogram was used. Concerning the calibration range of 5.0–50.0 μg/mL, the detector response was linear for organic acids whereas correlation coefficients exceeded 0.9992. The individual compounds were identified according to the time of retention and the method of standard addition.

### 2.6. Determination of Low Molecular Weight Saccharides Using High-Performance Liquid Chromatography

The presentation of individual carbohydrates (L-rhamnose, L-arabinose, D-fructose, D-glucose, xylose, trehalose, maltose, and saccharose) in appropriate extracts was analyzed using the HPLC equipment consisting of Thermo Scientific DionexUltimate 3000 coupled to a detector ERC RefractoMax 520, Ultimate 3000 autosampler, binary pump HPG-3xRS and solve selector valve HPG-30400RS (Waltham, MA, USA). The carbohydrates’ profile was measured using a Phenomenex Rezex RCM-Monosaccharide Ca^+2^ column (100 × 7.8 mm; 8 μm, Phenomenex, Torrance, CA, USA) in isocratic elution mode whereas redistilled water as a mobile phase with the rate of flow of about 0.4 mL/min for 15 min was applied. The injected volume was adjusted to 45 µL. The column chamber and cell of the RI detector were adjusted at 80 and 35 °C, respectively. Chromatograms with linear responses were recorded within the calibration ranges of 0.5–10.0 µg/mL with correlation coefficients exceeding 0.9990. Individual analytes were evaluated tentatively according to the time of retention for standards and the method of standard addition. Data signals were evaluated by LC ChromeleonTM 7.2 software (Thermo Scientific, Waltham, MA, USA) [25].

### 2.7. Effect of In Vitro Digestion Process on the Saccharide and Organic Acid Content

The amounts of each saccharide and organic acid that retained in the undigested portion of the tea leaves were expressed as retention factors (RF, %). The RF value was calculated using Equation (6):

RF = [CUWF × (100 − DMD)]/CNWF
(6)

where the appropriate values indicate the following: RF (the retention factor of the compound in the undigested part of the leaves, %), CUWF (the concentration of the compound in the undigested part of the leaves, mg/g), DMD (the dry matter digestibility value, %), and CNWF (the concentration of the compound in the raw form of the matcha, mg/g) [19].

### 2.8. Statistical Analysis

All analyses were replicated 3–5 times and their results were reported as mean ± standard deviation on a dry weight basis. The results of all analyses were statistically reported using a one-way analysis of variance (ANOVA). Subsequently, Tukey’s test was applied to identify the differences among means. The significance level of all statistical tests was set at 0.05.

## 3. Results and Discussion

### 3.1. Dry Matter, Ash Contents, and In Vitro Dry Matter Digestibility

The results of ash and dry matter contents and the dry matter digestibility values measured under in vitro conditions are shown in Table 1. The dry matter values ranged between 96.4 and 98.3%. According to Reg. No. 187, the dry matter content for green tea leaves must not be below 90% [26]. All matcha samples met this requirement. The obtained data may be aligned with data supported by Koláčková [19]. Regarding the regulation of the ash content in tea leaves, ISO 11287 stated minimum and maximum levels of 4 and 8%, respectively [27]. The ash values in matcha tea differed significantly between 5.05 and 6.66% and were in accordance with the regulation. It could be emphasized that Japanese matcha teas (Iswari, Natu) were measured to have a lower ash content than matcha tea samples originating from China (Organis, Allnature, Imbio). It was published that the ash amount should be below 5.54% to arrange a good quality of tea leaves [28].

Regarding the in vitro digestion process assay, matcha tea dry matter is digestible up to 67.3% during the digestion process in the stomach, and up to 85.9% when digested in both the stomach and the small intestine. Although data relating to the determination of the digestibility values for matcha tea leaves are scarce, our results might be compared to the research postulated by Koláčková [29].

### 3.2. Organic Acid Contents in Matcha Samples

Instead of catechins, amino acids and caffeine tea leaves contain organic acids and saccharides that interact in complex ways to form the unique taste of tea [13]. Phenolic substances, amino acids and xanthine alkaloids have already been described in the literature [19,29]; for this reason, the purpose of our study was to analyze the contents and digestibility of other analytes participating in the taste profile of matcha tea.

As can be seen in Table 2, the organic acids were assessed in the following order, according to their maximum amounts in matcha: citric acid (up to 44.8 mg/g) > malic acid (up to 32.2 mg/g) > oxalic acid (up to 4.46 mg/g) ≥ succinic acid (up to 4.44 mg/g).

The profile of individual organic acids in tea leaves is displayed in Figure S1. The organic acids most represented in the matcha are citric acid, in a concentration range of 9.01–44.8 mg/g, followed by malic acid. Our results agree with the study provided by Das [30], in which green tea leaves were analyzed. It has been proven that their concentration ranges are closely related to the time of shading, climate and harvesting conditions, fertilizers, and processing technology [17]. For example, the concentration of citric acid was measured in high levels in the leaves harvested at sunrise in May, and then in September [11]. The high concentrations of citric acid were measured in crude white tea leaves (4.78 mg/g), while succinic acid was not detected [17]. In contrast, oxalic and succinic acids are represented by one order lower in their concentration values. Our results agree with the studies presented by Shirai [11] and Morita and Tuji [31]. Although succinic acid is found in matcha tea in lower concentrations, it can contribute to the umami flavor as a taste enhancer together with glutamic and gallic acids, theogallin, and L-theanine [18,19,29]. The shading of green tea leaves was found to reduce the amount of quinic acid and increase the value of malic, citric, and oxalic acids [3,11,17,31]. Furthermore, quinic acid is predominantly stored in stems, not in leaves [8]. Due to this fact, it was not included in our research area.

Because matcha tea is consumed as an infusion suspension with ground green leaves, it is essential to determine the amounts of organic acids in both the native and undigested parts of the matcha leaves. The concentrations of organic acids measured after the digestion process simulated in the stomach and in both the stomach and small intestine are summarized in Table 3 and Table 4. These results were the basis for the calculation of retention factors (RF, %) for individual organic acids during the digestion process.

### 3.3. Low Molecular Weight Saccharide Contents in Matcha Samples

Focusing on the low molecular weight carbohydrates in matcha tea, scarce information has been reported. Table 5 shows the concentrations of the individual sugars measured in the native portion of matcha tea. D-fructose, xylose, maltose, and saccharose were not observed in all analyzed matcha tea samples, indicating their absence or their amounts were below their LOQ limit. In addition, Organis and Allnature matcha samples were poor in L-arabinose concentrations, but these were the only samples in which L-rhamnose occurred.

The profile shows that trehalose was the most abundant sugar in matcha tea (6.95–36.1 mg/g) (Figure S2). Research data on the trehalose content in matcha tea have not been previously addressed exhaustively in the scientific literature. However, it can be assumed that plants synthesize trehalose as a stress protectant. When carbon availability in plants under dark conditions is increased by the feeding of saccharose, the trehalose content rises [32]. The hypothesis of whether the concentration of trehalose in *Camellia sinensis* leaves also increases with the shading technique needs to be verified in the future. L-Arabinose was represented at a very low concentration only in Organis and Allnature matcha samples, while in the others it was present at concentrations one order of magnitude higher (5.86 to 8.20 mg/g). Different results were obtained in the determination of L-rhamnose, as it was detected only in Organis and Allnature samples (4.80 and 4.76 mg/g, respectively). Both L-rhamnose and L-arabinose are bonded in side chains of pectines which are cementing agents in the plant cell walls of tea leaves [33]. D-Glucose was determined in all samples and reached a concentration value of up to 4.14 mg/g. Regarding data based on D-fructose and D-glucose concentrations in green tea leaves, the D-fructose concentration (4.55 mg/g) was higher than the D-glucose value (3.61 mg/g) [12].

In contrast, Das [30] achieved the opposite results. The quantity of D-glucose was in the same magnitude range compared to our matcha samples, while D-fructose was not detected. As far as composition is concerned, several research studies, using a limited number of samples have revealed the identification and quantification of common carbohydrates with low molecular weight such as D-fructose, D-glucose, xylose, sucrose, and maltose [14,34]. In terms of target monosaccharides, concentrations of xylose (138 µg/g), D-fructose (4.16 mg/g), and D-glucose (3.77 mg/g) were evaluated in green tea leaves grown without shading techniques. In addition, saccharose (15.5 mg/g) and maltose (264 µg/g) as representatives of oligosaccharides were monitored [34].

Different results were published in the study provided by Shanmugavelan [14], when D-fructose, D-glucose, and saccharose reached concentrations in green tea leaves of 7.20, 6.80, and 7.10 mg/g, respectively, while maltose was not detected at all. Based on previous research studies, it can be stated that matcha tea contains relatively low concentrations of sugars that cannot negatively affect the daily calorie intake and consequently the glycemic index [12,15,35,36]. In addition, a significant increase in the glucosidase inhibitory activity by matcha tea was confirmed in both the gastric and intestinal phase of digestion [2].

In general, the nutritional characteristics mentioned to establish the quality of green tea are connected to its chemical constituents. The chemical characteristics of green tea (especially matcha tea) are affected by the shading time, the processing of the tea leaf, and other variables such as the environmental conditions, weather, season, and degree of leaf development [12]. Regarding saccharides, the monosaccharides L-rhamnose, L-arabinose, and D-glucose, and the oligosaccharide maltose in green tea are noticeably lower than those of fresh tea leaves [16]. It is a generally known fact that plants produce carbohydrates through photosynthesis. For this reason, it may be interesting to study the carbohydrate content depending on the shading technique with the subsequent production of matcha tea.

The amounts of individual saccharides assessed after the digestion process simulated in both the stomach and small intestine are summarized in Table 6 and Table 7. These results were used to evaluate retention factors (RF, %) for individual sugars during the digestion process.

### 3.4. Influence of the In Vitro Digestive Process on Organic Acid Retention in Matcha Tea

Our study was based on the hypothesis that the undigested parts of the matcha leaves move through the gastrointestinal tract to the large intestine, and thus assessed the effect of the in vitro digestibility of matcha tea on their organic acid and sugar values.

Regarding retention factor (RF, %) for each analyte, this research study focuses on the method to analyze the undigested part of matcha tea in order to determine the amounts of organic acids and low molecular weight saccharides after gastric and small intestinal digestion. To calculate the RF value, the amounts of analytes were evaluated in both the native and undigested portions of the matcha samples (Table 3, Table 4, Table 5, Table 6 and Table 7, Formula (6)). The RF factor indicates the concentration of the nutrient which remained in the undigested part after the digestion processes. The higher the RF value, the higher the concentration of the nutrient remained in the rest of the undigested part of the food, and thus was not available for absorption in the appropriate part of the gastrointestinal tract. In contrast, low RF substance should potentially be more easily absorbed in the appropriate part of the gastrointestinal tract [19].

**Figure 2:**
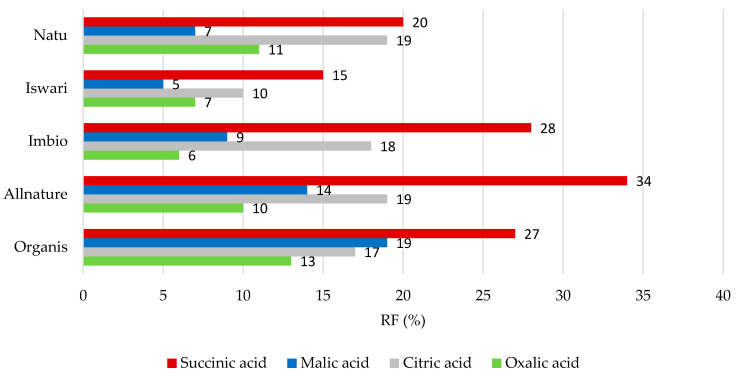
Remaining proportions expressed as retention factor (%) of organic acids after simulation of in vitro digestion in gastric phase.

**Figure 3:**
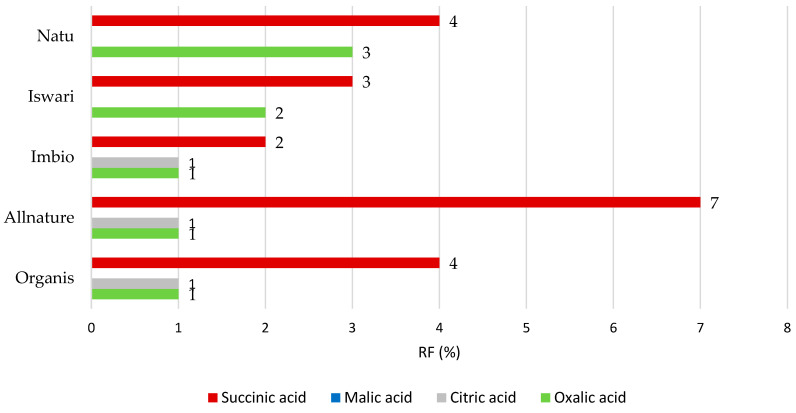
Remaining proportions expressed as retention factor (%) of organic acids after simulation of in vitro digestion in both gastric and intestinal phase.

The individual values of the retention factors for organic acids after the process of simulating digestion in the stomach and the small intestine are shown in Figure 2 and Figure 3. With regard to digestion in the stomach (as seen in Figure 2), succinic acid was the least-released organic acid with a RF value of 15 to 34%, followed by citric acid where the RF value reached 10–19%. It is evident that oxalic acid, with a retention factor value of 6 to 13%, is the most easily released from the matrix of the green tea leaf in this part of the digestive tract.

Focused on digestion in the stomach and small intestine (Figure 3), the highest RF value was obtained in the case of succinic acid where the RF value reached up to 7%. The order from least- to most-released organic acid is as follows: succinic > oxalic > citric > and malic acids. It should be emphasized that approximately 93% of succinic acid is theoretically available for absorption in the digestive tract, and up to 7% of its concentration remains bound in matcha tea leaves and passes into the large intestine. Conversely, malic acid is completely released from the matrix of the tea leaf after the digestion process (the RP value is 0%). In the measurement of oxalic acid, a retention factor of up to 3% was calculated, while for citric acid it was evaluated only for three out of five matcha tea samples. In concrete terms, citric acid was completely released from the matrix of the Natu and Iswari samples that originated in Japan.

Because there is a lack of bioavailability research on individual organic acid compounds analyzed in matcha tea, it is difficult to compare the results with other findings, such as those obtained in the native or undigested part of tea leaves. Matcha is known as a tea with a relatively high content of oxalic acid. Therefore, the drinking of this tea might lead to excessive intake of oxalic acid, which may increase the risk of kidney stone formation [37]. Furthermore, oxalic acid easily reacts with the Ca^2+^ ion, interfering with its absorption and decreasing its bioavailability. Considering the fact that the shade technique increases the amount of oxalic acid in green leaves which is important for the manufacturing of matcha tea [8,30], and that this acid shows a very low retention factor value (1–3%), it would be appropriate in the future to evaluate the recommended maximum daily intake of matcha tea for people sensitive to urinal stone formation.

Since low retention values have been recorded for organic acids—they are very easily released from the matrix of matcha tea leaves—we can suppose that they will perform a positive effect in inhibiting the proliferation of pathogenic bacteria by lowering the pH value in the intestine [17].

### 3.5. Effect of the In Vitro Digestibility on Saccharides Retention in Matcha

**Figure 4:**
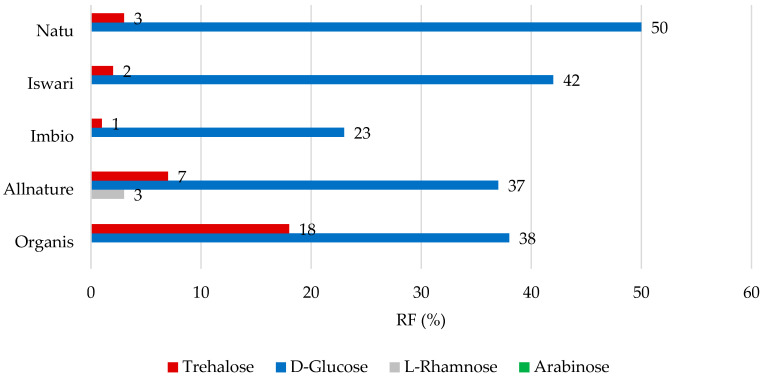
Remaining proportions expressed as retention factor (%) of saccharides after simulation of in vitro digestion in gastric phase.

**Figure 5:**
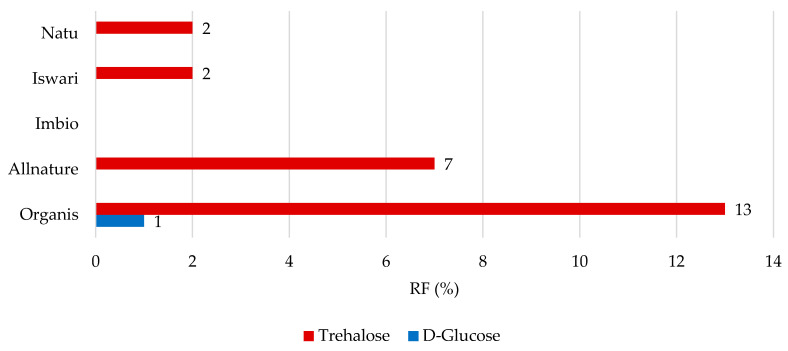
Remaining proportions expressed as retention factor (%) of saccharides after simulation of in vitro digestion in both gastric and intestinal phase.

Concerning low molecular weight saccharides, data comprising the RF values for individual analytes are shown in Figure 4 and Figure 5. After simulation in the gastric phase (Figure 4), D-glucose was retained in the matcha tea leaf in the highest proportions, with RF values of 23 to 50%, followed by trehalose (RF value between 1 and 18%). Only the Iswari tea sample showed a retention of 3% in the case of the saccharide L-rhamnose; this sugar was completely released from other tea samples. In the case of L-arabinose, the RF values reached 0%. It can be assumed that it was released entirely from the green leaves of matcha tea and may be potentially used in the intestine. Studies on the simulation of the digestion process of carbohydrates from the matrix of matcha tea leaves are not widespread. L-arabinose was shown to significantly alter the composition of microorganisms in the gut, particularly elevating Bifidobacterium under both in vivo and in vitro conditions [37]. Notably, it was published that L-arabinose shows the ability to reduce glucose absorption, and consequently, positively influence glycemic affect. This can be explained by its low digestion and low rate of absorption in the digestive tract [38]. The hypothesis that the L-arabinose released from matcha tea could also support these biological effects would require extensive studies in the future.

Although the highest RF values for D-glucose were found after gastric digestion, this statement cannot be applied to the simulation of the digestion process in the stomach and small intestine (Figure 5). D-glucose was completely released from green tea leaves, and the only 1% remaining was still captured in the Organis tea sample. On the contrary, trehalose was not completely released from matcha and even 13% of trehalose can still remain in tea leaves, which means that 87% of trehalose is available for digestion. Data concerning the bioaccessibility of saccharides from matcha tea leaves are misleading. The results can be compared with the bioaccessibility values of the total sugars of carbonated beverages, which ranged from 54.6 to 69.4%, whereas the total sugar content (consisting mainly of D-glucose and D-fructose) ranged from 9.23 to 13.1 g/100 mL [39]. Compared to most sugars, trehalose is composed of a 1,1 linkage of two molecules of D-glucose, which is not easily hydrolyzed by acids and is more stable in the acid and basic ranges of the pH scale and at a higher temperature. In addition, this type of glycosidic bond is not destroyed by α-glucosidase. When ingested, it is hydrolyzed using the enzymes in the small intestine [40]. This could explain its retention value in the tea leaf matrix. Interestingly, it seems that trehalose may not be completely hydrolyzed in the small intestine and may continue to the large intestine.

## 4. Conclusions

This research work provides data on the organic acid, low molecular weight carbohydrates, and dry matter digestibility values in matcha teas and their undigested portions. This unique study also evaluates the effect of the in vitro digestion process on retention factors of individual analytes. The organic acid contents were established in this order: citric acid (up to 44.8 mg/g) > malic acid > oxalic acid ≥ succinic acid (up to 4.44 mg/g). Taking into account the low molecular weight carbohydrates in native matcha samples, the sequence of the highest amounts is as follows: trehalose > L-arabinose > L-rhamnose > D-glucose, while L-rhamnose was evaluated only in two of the five samples, and D-fructose, xylose, maltose, and saccharose were not detected in any of the matcha teas. It can be assumed that plants synthesize trehalose as a stress protectant, and it may be interesting in the future to study the carbohydrate content depending on the shading technique. The highest retention factor (RF) after in vitro digestion in the stomach was found for succinic acid (15–34%), followed by citric, malic, and oxalic acids (6–13%). Regarding gastric and intestinal digestion, malic acid was completely released from the matrix of the tea leaves; contrarily, succinic acid (RF up to 7%) was still bounded in matcha tea leaves and theoretically it should pass into the large intestine. To evaluate the RF value for saccharides after gastric digestion, D-glucose (23–50%) followed by trehalose (1–18%) was retained in the matcha tea leaf in the highest proportions, while L-arabinose was completely released. D-glucose was completely released from green tea leaves only after simulating both gastric and intestinal phases of digestion; in contrast, trehalose was not completely released from matcha, and even 13% of trehalose can still remain in tea leaves, and it theoretically seems to pass to the large intestine.

Results can facilitate a better understanding of the chemical composition, reinforcing consumers’ perception of matcha tea as a health-promotion beverage prepared in the form of the whole leaf parts. In the measurement of oxalic acid, considering that the process of shading tea leaves increases the concentration of this acid and its RF value is too small, it would be appropriate in the future to evaluate the recommended maximum daily intake of matcha tea for people sensitive to the formation of urinal stones, for example. In addition, for the industry, our knowledge could mainly contribute to the identification of matcha tea quality markers in the future, and the elucidation of the chemical reactions that occur during the shading processing of green tea leaves into matcha tea.

## Figures and Tables

**Table 1:** Results of the ash, dry matter, and DMD value.

Matcha	Dry Matter (%)	Ash (%)	DMD (%)	
			Gastric Digestion	Gastric and Intestinal Digestion
Organis	96.4 ± 0.1 ^a^	6.02 ± 0.04 ^a^	66.5 ± 1.0 ^a^	85.9 ± 2.1 ^a^
Allnature	96.6 ± 0.2 ^a,c^	6.38 ± 0.08 ^b^	64.4 ± 0.9 ^b^	84.7 ± 1.0 ^a,b^
Imbio	97.3 ± 0.2 ^b,c^	6.66 ± 0.01 ^c^	67.3 ± 2.0 ^c^	83.7 ± 2.0 ^b^
Iswari	96.9 ± 0.4 ^c^	5.05 ± 0.01 ^d^	64.5 ± 2.2 ^b^	83.7 ± 2.0 ^b^
Natu	98.3 ± 0.2 ^d^	5.34 ± 0.05 ^e^	58.1 ± 1.3 ^d^	82.4 ± 1.1 ^c^

All results are presented on a dry matter basis as means ± SD; n = 3 (the mean of five measurements). Means within a column with various superscripts letter show a significant difference (*p* < 0.05). DMD—Dry matter digestibility value.

**Table 2:** Organic acid contents determined in the native form of matcha.

Matcha	Oxalic Acid (mg/g)	Citric Acid (mg/g)	Malic Acid (mg/g)	Succinic Acid (mg/g)
Organis	4.46 ± 0.18 ^a^	33.2 ± 1.0 ^a^	27.7 ± 0.8 ^a^	4.44 ± 0.12 ^a^
Allnature	4.27 ± 0.12 ^b^	24.1 ± 1.0 ^b^	25.9 ± 1.2 ^a^	2.70 ± 0.23 ^b^
Imbio	3.39 ± 0.12 ^c^	9.01 ± 0.13 ^c^	20.3 ± 1.0 ^b^	0.98 ± 0.03 ^c^
Iswari	2.42 ± 0.11 ^d^	26.2 ± 1.1 ^d^	32.2 ± 1.5 ^c^	2.86 ± 0.13 ^b^
Natu	1.96 ± 0.12 ^e^	44.8 ± 1.3 ^e^	29.2 ± 1.4 ^c,a^	3.41± 0.11 ^d^

All results are presented on a dry matter basis as means ± SD; n = 5 (the mean of five measurements). Means within a column with various superscripts letter show a significant difference (*p* < 0.05).

**Table 3:** Organic acid contents determined in the matcha sample after gastric digestion.

Matcha	Oxalic Acid (mg/g)	Citric Acid (mg/g)	Malic Acid (mg/g)	Succinic Acid (mg/g)
Organis	1.73 ± 0.08 ^a^	16.9 ± 0.8 ^a^	15.9 ± 0.5 ^a^	3.61 ± 0.02 ^a^
Allnature	1.15 ± 0.02 ^b^	13.0 ± 0.3 ^b^	10.4 ± 0.2 ^b^	2.59 ± 0.02 ^b^
Imbio	0.61 ± 0.03 ^c^	4.93 ± 0.12 ^c^	5.44 ± 0.12 ^c^	0.85 ± 0.07 ^c^
Iswari	0.47 ± 0.05 ^d^	7.69 ± 0.20 ^d^	4.21 ± 0.11 ^d^	1.20 ± 0.04 ^d^
Natu	0.52 ± 0.03 ^e^	19.8 ± 0.6 ^e^	4.60 ± 0.10 ^e^	1.62 ± 0.06 ^e^

All results are presented on a dry matter basis as means ± SD, n = 5 (the mean of five measurements). Means within a column with various superscripts letter show a significant difference (*p* < 0.05).

**Table 4:** Organic acid contents determined in matcha after gastric and intestinal digestion.

Matcha	Oxalic Acid (mg/g)	Citric Acid (mg/g)	Malic Acid (mg/g)	Succinic Acid (mg/g)
Organis	0.43 ± 0.07 ^a^	1.90 ± 0.02 ^a^	0.54 ± 0.02	1.33 ± 0.05 ^a^
Allnature	0.29 ± 0.03 ^b^	0.91 ± 0.05 ^b^	n.d.	1.16 ± 0.10 ^b^
Imbio	0.20 ± 0.03 ^c^	0.69 ± 0.02 ^c^	n.d.	0.15 ± 0.02 ^c^
Iswari	0.30 ± 0.04 ^b^	0.56 ± 0.03 ^d^	n.d.	0.50 ± 0.01 ^d^
Natu	0.31 ± 0.02 ^b^	0.19 ± 0.01 ^e^	n.d.	0.84 ± 0.02 ^e^

All results are presented on a dry matter basis as means ± SD, n = 5 (the mean of five measurements). Means within a column with various superscripts letter show a significant difference (*p* < 0.05). LOQ (limit of quantification): malic acid (0.002 mg/g). n.d.—Not detected.

**Table 5:** Saccharide contents determined in native form of matcha.

Matcha	L-Arabinose (mg/g)	L-Rhamnose (mg/g)	D-Glucose (mg/g)	D-Fructose (mg/g)
Organis	0.76 ± 0.04 ^a^	4.80 ± 0.40 ^a^	1.74 ± 0.15 ^a^	n.d.
Allnature	0.90 ± 0.02 ^b^	4.76 ± 0.25 ^a^	1.67 ± 0.11 ^a^	n.d.
Imbio	8.20 ± 0.27 ^c^	n.d.	4.14 ± 0.08 ^b^	n.d.
Iswari	5.86 ± 0.21 ^d^	n.d.	2.08 ± 0.14 ^c^	n.d.
Natu	7.88 ± 0.43 ^e^	n.d.	2.25 ± 0.07 ^c^	n.d.
	**Xylose (mg/g)**	**Trehalose (mg/g)**	**Maltose (mg/g)**	**Saccharose (mg/g)**
Organis	n.d.	6.95 ± 0.32 ^a^	n.d.	n.d.
Allnature	n.d.	11.2 ± 0.4 ^b^	n.d.	n.d.
Imbio	n.d.	36.1 ± 0.9 ^c^	n.d.	n.d.
Iswari	n.d.	25.6 ± 0.6 ^d^	n.d.	n.d.
Natu	n.d.	33.1 ± 1.1 ^e^	n.d.	n.d.

All results are presented on a dry matter basis as means ± SD, n = 5 (the mean of five measurements). Means within a column with various superscripts letter show a significant difference (*p* < 0.05). LOQ: D-fructose, maltose, and saccharose (0.001 mg/g); xylose (0.002 mg/g). n.d.—Not detected.

**Table 6:** Saccharide contents determined in the matcha sample after gastric digestion.

Matcha	L-Arabinose (mg/g)	L-Rhamnose (mg/g)	D-Glucose (mg/g)	D-Fructose (mg/g)
Organis	n.d.	n.d	1.96 ± 0.04 ^a^	n.d.
Allnature	n.d.	0.43 ± 0.08	1.72 ± 0.10 ^b^	n.d.
Imbio	n.d.	n.d.	2.87 ± 0.10 ^c^	n.d.
Iswari	n.d.	n.d.	2.48 ± 0.01 ^d^	n.d.
Natu	n.d.	n.d.	2.66 ± 0.02 ^e^	n.d.
	**Xylose (mg/g)**	**Trehalose (mg/g)**	**Maltose (mg/g)**	**Saccharose (mg/g)**
Organis	n.d.	3.65 ± 2.21 ^a^	n.d.	n.d.
Allnature	n.d.	2.31 ± 1.57 ^a^	n.d.	n.d.
Imbio	n.d.	1.49 ± 0.89 ^a^	n.d.	n.d.
Iswari	n.d.	1.77 ± 0.58 ^a^	n.d.	n.d.
Natu	n.d.	2.75 ± 0.39 ^a^	n.d.	n.d.

All results are presented on a dry matter basis as means ± SD, n = 5 (the mean of five measurements). Means within a column with various superscripts letter show a significant difference (*p* < 0.05). LOQ: L-arabinose, D-fructose, maltose, and saccharose (0.001 mg/g); xylose and L-rhamnose (0.002 mg/g). n.d.—Not detected.

**Table 7:** Saccharide contents determined in the matcha sample after gastric and intestinal digestion.

Matcha	Arabinose (mg/g)	L-Rhamnose (mg/g)	D-Glucose (mg/g)	D-Fructose (mg/g)
Organis	n.d.	n.d.	0.14 ±0.01	n.d.
Allnature	n.d.	n.d.	n.d.	n.d.
Imbio	n.d.	n.d.	n.d.	n.d.
Iswari	n.d.	n.d.	n.d.	n.d.
Natu	n.d.	n.d.	n.d.	n.d.
	**Xylose (mg/g)**	**Trehalose (mg/g)**	**Maltose (mg/g)**	**Saccharose (mg/g)**
Organis	n.d.	6.60 ± 0.18 ^a^	n.d.	n.d.
Allnature	n.d.	4.79 ± 0.31 ^a^	n.d.	n.d.
Imbio	n.d.	n.d.	n.d.	n.d.
Iswari	n.d.	4.21 ± 0.21 ^b^	n.d.	n.d.
Natu	n.d.	3.94 ± 0.22 ^b^	n.d.	n.d.

All results are presented on a dry matter basis as means ± SD, n = 5 (the mean of five measurements). Means within a column with various superscripts letter show a significant difference (*p* < 0.05). LOQ: L-arabinose, D-glucose, D-fructose, trehalose, maltose, and saccharose (0.001 mg/g); xylose and L-rhamnose (0.002 mg/g). n.d.—Not detected.

## Data Availability

Data is contained within the article. The original contributions presented in this study are included in the article/Supplementary Materials. Further inquiries can be directed to the corresponding author.

## Supplementary Materials

The following supporting information can be downloaded at: https://www.mdpi.com/article/10.3390/nu16234058/s1, Figure S1: Individual profile of organic acids in matcha teas; Figure S2: Individual profile of free saccharides in matcha teas.
