# Supplementary material for: The Release of Organic Acids and Low Molecular Weight Carbohydrates from Matcha Tea After In Vitro Digestion
Source: Nutrients. 2024 Nov 26;16(23):4058. doi: 10.3390/nu16234058 (PMC11643872; doi:10.3390/nu16234058)
Supplement: Supplementary file 1 [file nutrients-16-04058-s001.zip › nutrients-3319830-supplementary.pdf]

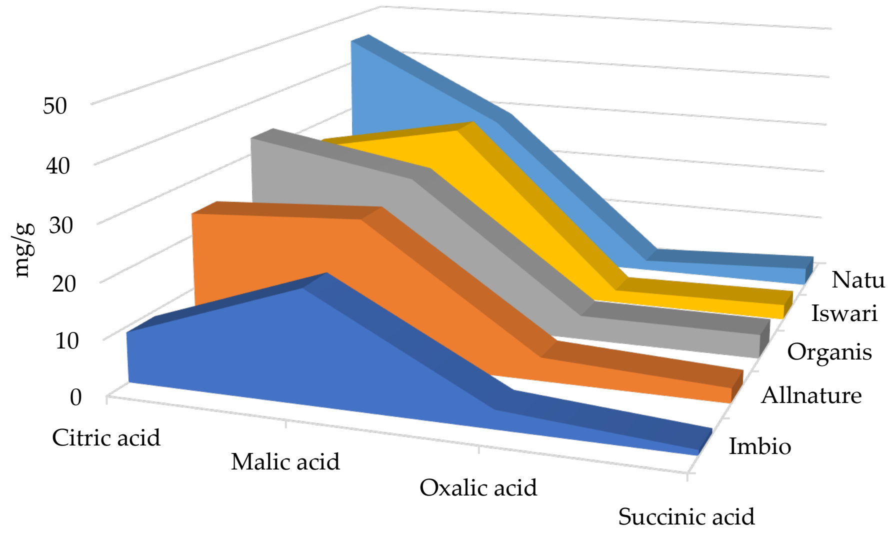

Figure S1. Individual profile of organic acids in matcha teas.

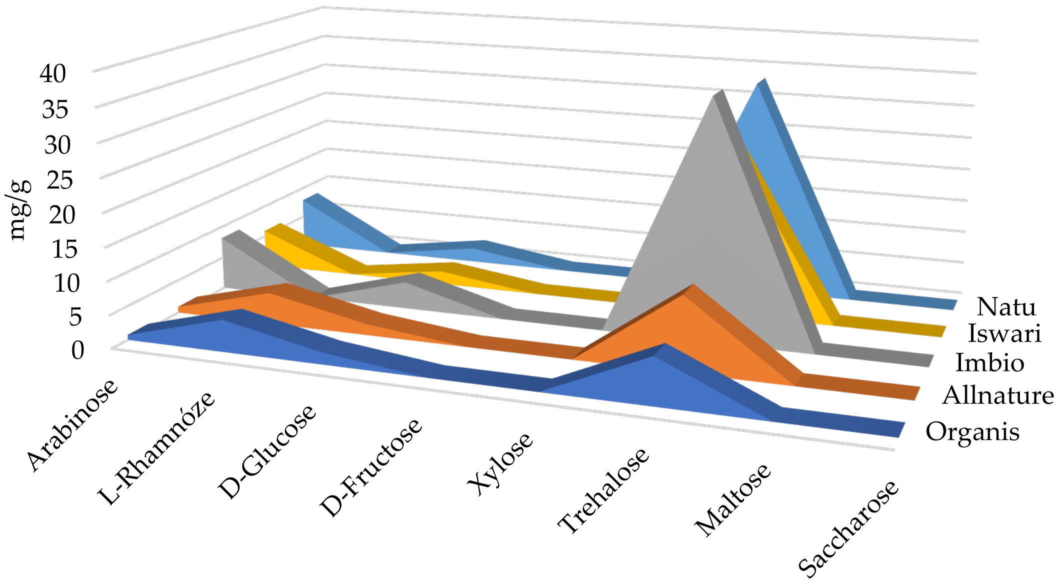

Figure S2. Individual profile of free saccharides in matcha teas.
